# Efficient simulation of potential energy operators on quantum hardware: a study on sodium iodide (NaI)

**DOI:** 10.1038/s41598-024-60605-6

**Published:** 2024-05-11

**Authors:** Mostafizur Rahaman Laskar, Atanu Bhattacharya, Kalyan Dasgputa

**Affiliations:** 1 IBM Research, Bangalore, India; 2 Department of Chemistry, GITAM, Visakhapatnam, India; 3 G. S. Sanyal School of Telecommunications, Indian Institute of Technology Kharagpur, Kharagpur, India

**Keywords:** Hamiltonian simulation, Quantum computing, First quantization, Potential energy, Basis encoding, Quantum simulation, Quantum chemistry

## Abstract

This study introduces a conceptually novel polynomial encoding algorithm for simulating potential energy operators encoded in diagonal unitary forms in a quantum computing machine. The current trend in quantum computational chemistry is effective experimentation to achieve high-precision quantum computational advantage. However, high computational gate complexity and fidelity loss are some of the impediments to the realization of this advantage in a real quantum hardware. In this study, we address the challenges of building a diagonal Hamiltonian operator having exponential functional form, and its implementation in the context of the time evolution problem (Hamiltonian simulation and encoding). Potential energy operators when represented in the first quantization form is an example of such types of operators. Through systematic decomposition and construction, we demonstrate the efficacy of the proposed polynomial encoding method in reducing gate complexity from $\mathcal {O}(2^n)$ to $\mathcal {O}\left( \sum _{i=1}^{r} {} ^nC_r \right)$ (for some $r\ll n$). This offers a solution with lower complexity in comparison to the conventional Hadamard basis encoding approach. The effectiveness of the proposed algorithm was validated with its implementation in the IBM quantum simulator and IBM quantum hardware. This study demonstrates the proposed approach by taking the example of the potential energy operator of the sodium iodide molecule (NaI) in the first quantization form. The numerical results demonstrate the potential applicability of the proposed method in quantum chemistry problems, while the analytical bound for error analysis and computational gate complexity discussed, throw light on issues regarding its implementation.

## Introduction

The development of accurate, efficient methods for the electronic structure of molecules, solids, and clusters has been a topic of immense interest in chemistry, physics and materials science. Electronic-structure calculations generally use an occupation number representation approach (also known as second quantization)^1–3^. The choice of basis set decides the accuracy of a calculation. Different basis sets are used to deal with different systems. Some of the notable ones include the plane waves (PW) or atom-centered localized basis sets such as Slater-type orbitals (STO) and Gaussian-type orbitals (GTO). In general, all second quantization approaches have a persistent problem arising due to the basis-set incompleteness. One of the first and most important decisions that need to be made by a physicist or chemist while planning such a calculation is the choice of basis set.

Another approach to electronic structure theory calculations is to use the spatial grid method (called first quantization). Several authors have discussed the spatial grid method for the first-principles electronic-structure calculations^4,5^, and various developments and applications have shown that the real-space method is perhaps the most suitable approach for systems with unprecedentedly large sizes^5,6^. In particular, the potential operator is diagonal in coordinate space and as a result, the spatial grid method can potentially remove the inherent problem arising due to the basis-set incompleteness in the basis-set approaches. Furthermore, spatial grids are easily amenable to the so-called linear-scaling methods (the computer time required for the calculation is linearly proportional to the number of atoms present)^7^. Quantum mechanical problems that deal with extensive systems, such as drug discovery, discovery of efficient catalysts or problem of protein folding, can be easily solved by using a linear-scaling method, which is hard to achieve using a basis set approach. While at face value, the spatial grid method appears to demand a larger number of grids to achieve physically meaningful results, the effective grid can be reduced significantly by introducing higher-order and multi-grid techniques. Furthermore, from a quantum computing perspective, we envision that the quantum computer can easily fulfil the requirement of a large grid. With this in mind, we have explored the feasibility of implementing the grid-based method to express potential energy curves in the quantum computing construct in our present work.

**Figure 1:**
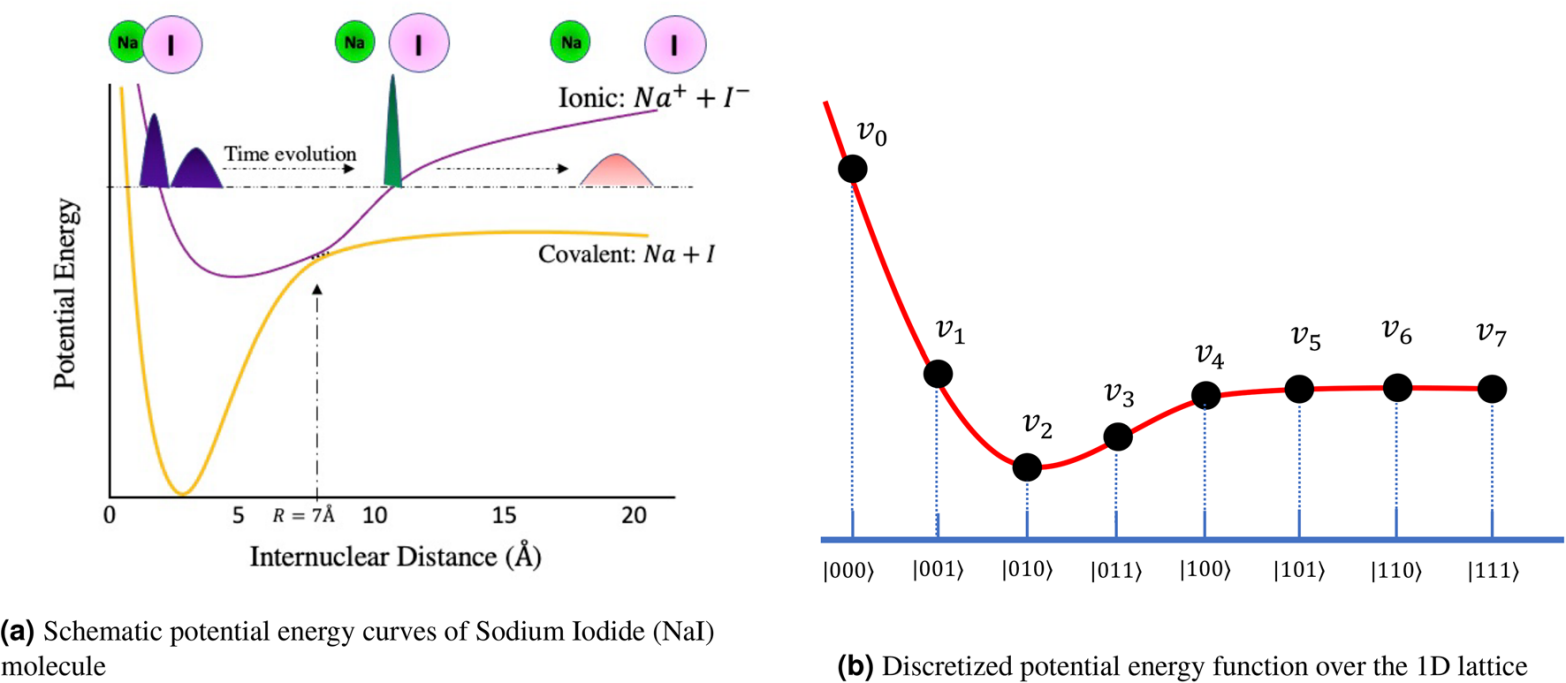
Potential Energy curves of NaI and the discretized potential energy function is shown as a function of 1D grid points of space variable $\hat{x}$: (**a**) denotes the potential energy curve of NaI, and (**b**) represents the discretized potential energies and its encoding in 3 qubits.

Over the years, researchers have employed diverse theoretical and computational methods to explore and represent the potential energy landscapes and understand their influence on molecular structure and reactivity. Recent state-of-the-art literature highlights the potential of machine learning techniques, such as neural network potential energy surfaces, to efficiently approximate complex potential energy landscapes^8–10^. Furthermore, quantum algorithms have been developed for grid-based variational time evolution and threshold gate-based quantum simulation, enabling accurate simulations of quantum systems with reduced computational resources^11–15^. However, due to the difficulty of achieving chemical accuracy and due to the curse of dimensionality in many chemical dynamics studies, classical techniques become intractable. To cope with these limitations, quantum algorithms may be used to show potential applications in this regime. The advantage that Quantum computing gives is in the way the matrix exponential is easily realized by decomposing them into strings of Pauli matrices. For non-commuting Hamiltonian terms, the Trotter-Suzuki method may be applied for the individual terms^15^. However, present-day Quantum computers face limitations as the number of trotter terms increases. These methods are mainly envisaged for fault-tolerant quantum systems.

The limitations that noisy present-day quantum systems challenge us with make it all the more important to develop quantum algorithms for Hamiltonian (potential and kinetic energy) operators with reduced computational complexity^16^. As the system size increases, the computational resources required for accurate simulations grow exponentially, leading to the “curse of dimensionality that hampers exact simulations for large systems^17,18^. Novel quantum Hamiltonian encoding algorithms are needed to overcome these limitations. Efficient encoding schemes that reduce the gate complexity while preserving the accuracy of the simulation will be instrumental in making potential energy simulations accessible on near-term quantum hardware^19–21^. By mitigating the computational challenges, these advancements will pave the way for furthering our understanding of atomic behaviour and unlocking the full potential of quantum technologies in various scientific applications^11,22^. In Figure 1a, the potential energy curve of Sodium Iodide (NaI) as a function of inter-nuclear separation is shown. Snapshots of a time-evolving wave packet created on the excited potential energy curve of NaI are also schematically shown. In our work, we have tested our newly proposed polynomial encoding quantum algorithm using the NaI ionic and covalent potentials.

In this work, our research contribution is twofold. First, we extend the idea of Walsh series approximation of a given potential energy function without ancillary qubit as demonstrated in Ref.^23^. Secondly, we propose a polynomial encoding algorithm for diagonal Hamiltonian simulation, considering the first quantization form for large systems. The specific contributions of this article are given as follows.

- We show how Hadamard basis encoding can be applied to encode a diagonal energy operator arbitrarily close to the actual function. The computation of the coefficients of the Hadamard basis expansion of the function is also shown in reference to its implementation using elementary quantum gates (i.e., $\textbf{Z}$ and $\textbf{I}$ operators here).
- We have proposed a conceptually new algorithm, namely polynomial encoding, considering the first quantization level for large systems. The $r^{th}$ order approximation using the polynomial basis function is discussed with its implementation shown for 2 to 4-qubit quantum systems.
- The quantum circuit optimization is shown for the polynomial encoding scheme compared to the Hadamard basis encoding scheme. Circuit implementation of the given functions (an exponential function associated with the potential energy operator of NaI as a case study) in qiskit^24^ are provided.
- The error bound is shown for the time evolution operator embedding potential energy function with the polynomial basis encoding. The gate complexity analysis for the polynomial encoding scheme shows a complexity reduction from $2^n$ to $\displaystyle \sum _{i=1}^{r} {}^nC_r$. It gives significant resource optimization for the case $r<<n$ for encoding potential energy with a high sample size (sample size $N=2^n$).
- We have provided experimental results for the NaI molecule after encoding the time evolution operation (due to the potential energy function) on IBM quantum Hardware. The simulator-based and Hardware-based fidelity responses have been measured, in addition to the construction (of the unitary operator) and reconstruction of the potential energy operator.

## Hamiltonian encoding of potential energy

In the realm of non-relativistic physics, the Hamiltonian governs the intricate behaviour of particles, such as electrons, as they interact with the external potential of other particles, notably the positively charged nuclei. This intricate interplay is elegantly described within the Born-Oppenheimer approximation, as represented by

1 $$\begin{aligned} \textbf{H} = -\sum _{i} \frac{\nabla ^2_i}{2} - \sum _{i,j} \frac{q_j}{\vert R_j - r_i \vert } + \sum _{i<j} \frac{1}{r_i - r_j} + \sum _{i<j} \frac{q_i q_j}{R_i - R_j}. \end{aligned}$$

Here, $\textbf{H}_1=-\sum _{i} \frac{\nabla ^2_i}{2}$ is the kinetic energy term, $\textbf{H}_2=- \sum _{i,j}\frac{q_j}{\vert R_j -r_i \vert }$ denotes the potential energy where $q_j$ are charges of the nuclei, $R_j$ and $r_i$ are positions of the nuclei and electrons respectively; the term $\textbf{H}_3=\sum _{i<j}\frac{1}{r_i-r_j}$ denotes the electron-electron repulsion potential term, and $\textbf{H}_4=\sum _{i<j}\frac{q_iq_j}{R_i-R_j}$ is a constant term. The effective Hamiltonian is simplified as $\textbf{H} = \textbf{K}(\hat{p}) + \textbf{V}(\hat{x})$, where $\textbf{K}(\hat{p})$ represents the discretized kinetic energy operator as a function of momentum ($\hat{p}$), and the term $\textbf{V}(\hat{x})$ denotes the potential energy expressed in space coordinates ($\hat{x}$). Note that we assume all energy terms (except for the kinetic energy part) as part of the potential energy operator, as given in (2). We use discretization techniques to make the differential form (1) implementable on a digital computer^13,25^.

2 $$\begin{aligned} \textbf{V} = -\sum _{i,j} \frac{q_j}{\vert R_j - r_i \vert } + \sum _{i<j} \frac{1}{r_i - r_j} + \sum _{i<j} \frac{q_i q_j}{R_i - R_j}. \end{aligned}$$

The time-dependent Schrödinger’s equation, known as TDSE, describes how a quantum system evolves over time. It is represented as $i\hbar \frac{\partial }{\partial t}\mathinner {|{\Psi (t)}\rangle } = \hat{H}\mathinner {|{\Psi (t)}\rangle }.$ Here, the Hamiltonian operator, denoted as $\hat{H}$, consists of two parts: potential energy and kinetic energy. The evolution of the system under $\hat{H}$ can be expressed as $\mathinner {|{\Psi (t)}\rangle } = e^{-i\hat{H}t/\hbar }\mathinner {|{\Psi (0)}\rangle }$.

The potential energy affects the wave-function of the quantum system as distributed over the spatial dimensions. The state $\mathinner {|{\Psi (t)}\rangle }$ is like a vector state, with each component indicating the probability of finding the quantum system at a specific point in a 1D or 2D grid. For simplicity, let’s focus on a 1D grid, where each point corresponds to a computational basis state. If we only consider the potential energy, we can write $\hat{H}$ as $\textbf{V}(\hat{x})$, and the state evolves due to the operation of $e^{-i\textbf{V}(\hat{x})t/\hbar }$ on the state vector $\mathinner {|{\Psi (t)}\rangle }$. If the potential function is well-behaved (continuous and square-integrable) at every point on the grid, it can be represented as a diagonal matrix. The exponential of such a matrix is also diagonal. When we work with atomic units and set $\hbar$ to 1, we obtain the simplified equation $\mathinner {|{\Psi (t)}\rangle } = e^{-i\textbf{V}(\hat{x})t}\mathinner {|{\Psi (0)}\rangle }$. Considering the first quantization level, we treat the matrix $e^{-i\textbf{V}(\hat{x})t}$ as a diagonal unitary matrix.

We have represented the 1-dimensional lattice variable as a uniform grid denoted by the computational basis states of the system. These states range from $\mathinner {|{0}\rangle }^{\otimes n}$ to $\mathinner {|{1}\rangle }^{\otimes n}$ in an $n$-qubit system. The leftmost bit represents the most significant qubit (msqb), while the rightmost bit represents the least significant qubit (lsqb). If there are *N* number of classical lattice points over which the potential energy operator acts, we will need $n=\lceil \log N \rceil$ qubits to represent them.

*Note:* We have shown the potential energy function curve of NaI as a function of 1D grid points of space variable $\hat{x}$, as illustrated in Figure 1a. As an example, we take $N=8$ grid points, which require $\lceil \log N \rceil =3$ qubits to represent them. The slope of the function is stiff in the beginning (e.g., $\mathinner {|{000}\rangle }, \mathinner {|{001}\rangle }, \mathinner {|{010}\rangle }$), whereas the slope is gentle for the functional values $v_4, \dots , v_7$ corresponding to basis states $\mathinner {|{100}\rangle }, \dots , \mathinner {|{111}\rangle }$. In the quantum encoding method (as discussed next), we represent the discretized potential energy values $v_i$ for $i=0, 1\dots , 7$ as a diagonal matrix $\textbf{V}(\hat{x})$ given by

3 $$\begin{aligned} \textbf{V}(\hat{x})= \begin{bmatrix} v_0&{}&{}&{}&{}&{}&{}&{}\\ &{}v_1&{}&{}&{}&{}&{}&{}\\ &{}&{}v_2&{}&{}&{}&{}&{}\\ &{}&{}&{}v_3&{}&{}&{}&{}\\ &{}&{}&{}&{}v_4&{}&{}&{}\\ &{}&{}&{}&{}&{}v_5&{}&{}\\ &{}&{}&{}&{}&{}&{}v_6&{}\\ &{}&{}&{}&{}&{}&{}&{}v_7\\ \end{bmatrix}. \end{aligned}$$

As our objective is to design a unitary operator approximately close to $e^{-iV(x)t}$, we may parameterize the potential energy values with the parameters $\theta _i=v_i \times t$ for time *t*, and $i=0, 1\dots , 7$. The parameters give us the angles for the single and multi-qubit phase gates, which are then used to encode the time evolution operation. The details of the slope of the curve related with the internuclear distance of NaI is given in^26^. The implementation of the kinetic part of the propagator has been discussed in the recent work^27^.

### Hadamard basis encoding

To encode our potential function into basis functions that can be encoded in a quantum circuit, we use the Hadamard transform, or the Walsh-Hadamard transform^28^. The matrix generating the Hadamard functions can be obtained by taking the tensor products of Hadamard matrices, $H^{\otimes n}$. The Hadamard basis functions approximate a given function defined over an *n* qubit system with $2^n$ such basis functions. The factor $\frac{1}{\sqrt{2^n}}$ ensures that the bases are orthonormal and the corresponding matrix is unitary. The potential function over the lattice points can be represented as a linear combination of the bases as $f(x) = \sum _{j=1}^{2{}^n} c_j b_j(x)$, where *x* denotes the lattice points. The coefficient $c_j \in \mathbb {R}$ can be obtained by taking the inner product of the function *f*(*x*) with the basis $b_j(x)$ as $c_j = b_j(x)^T f(x)$. The basis $b_j$ is the *j*th column of the Hadamard matrix $H^{\otimes n}$.

The diagonal matrix $\textbf{V}(\hat{x})$, as given in (3), can be represented in Hadamard bases with the decomposition, $\textbf{V}(\hat{x}) = \sum _{j=1}^{2{}^n} c_j\textbf{B}_j$, where every $\textbf{B}_j$ is a diagonal matrix with $b_j$ as its principal diagonal. The corresponding time evolution operator can be written as $e^{-i\textbf{V}(\hat{x})t} = e^{-it\sum c_j\textbf{B}_j}$. Since diagonal matrices commute, we can write $e^{-i\textbf{V}(\hat{x})t} = \displaystyle \prod _{i=1}^{2{}^n} e^{-it c_j\textbf{B}_j} = \displaystyle \prod _{i=1}^{2{}^n} e^{-i\theta _j\textbf{B}_j}$, where $\theta _j = c_jt$. The parameter $\theta$ can be used to prepare the quantum circuit. It can be shown that the diagonal matrices represented by $\textbf{B}_j$s can be obtained by using tensor product combinations of Pauli $\textbf{Z}$ and $\textbf{I}$ matrices. The combination of $\textbf{Z}$ and $\textbf{I}$ follows the binary number progression ($000\dots$ to $111\dots$, all zeros to all ones) with $\textbf{I}$ corresponding to 0 and $\textbf{Z}$ corresponding to 1. The leftmost column or the leftmost basis of the Hadamard functions matrix corresponds to all zeros, and the rightmost column corresponds to all ones. Table 1 illustrates the idea for a 3-qubit system.

**Table 1:** Hadamard basis matrices and Pauli $\textbf{Z}$ and *I* equivalence.

Sl. No.	Basis matrix	Binary exp	*I* and $\textbf{Z}$ combination
1	$\textbf{B}_1$	000	$\textbf{I}\otimes \textbf{I}\otimes \textbf{I}$
2	$\textbf{B}_2$	001	$\textbf{I}\otimes \textbf{I}\otimes \textbf{Z}$
3	$\textbf{B}_3$	010	$\textbf{I}\otimes \textbf{Z}\otimes \textbf{I}$
4	$\textbf{B}_4$	011	$\textbf{I}\otimes \textbf{Z}\otimes \textbf{Z}$
5	$\textbf{B}_5$	100	$\textbf{Z}\otimes \textbf{I}\otimes \textbf{I}$
6	$\textbf{B}_6$	101	$\textbf{Z}\otimes \textbf{I}\otimes \textbf{Z}$
7	$\textbf{B}_7$	110	$\textbf{Z}\otimes \textbf{Z}\otimes \textbf{I}$
8	$\textbf{B}_8$	111	$\textbf{Z}\otimes \textbf{Z}\otimes \textbf{Z}$

$\textbf{B}_1$ is the diagonal matrix having the basis $b_1$ (the left most basis) in the diagonal. It corresponds to the binary number 000. This matrix can then be constructed by taking the tensor product $\textbf{I}\otimes \textbf{I}\otimes \textbf{I}$. Similarly, for the rest, with the final basis matrix $\textbf{B}_8$ having the basis $b_8$ in the diagonal. This corresponds to 111 in the binary number progression. This matrix can be created by doing the tensor product $\textbf{Z}\otimes \textbf{Z}\otimes \textbf{Z}$. $\textbf{B}_j$, thus, can be expressed in the form of *Z* and *I* matrices as given in Table 1, scaled by a factor of $\frac{1}{\sqrt{N}}$. The matrix exponential $e^{-i\theta _j \textbf{B}_j}$, expressed as an exponential of a tensor product of $\textbf{Z}$ and $\textbf{I}$ matrices, can be implemented in a quantum circuit by a combination of cascaded CNOT gates and a phase gate $R_z (2\theta )$. More on the Hadamard encoding circuit and gate counts can be found in Ref.^29^.

The upper bound on the total number of CNOT gates required for the *n* qubit quantum circuit using the Hadamard basis encoding method is given by

4 $$\begin{aligned} n_{CNOT} = \sum _{r=2}^{n} {}^nC_r 2(r-1). \end{aligned}$$

### Proposed polynomial basis encoding

We propose a conceptually novel Hamiltonian encoding procedure using a parameterized approach for an approximate time evolution with reduced computational complexity. In this procedure, we perform a polynomial fitting of the actual potential energy curve up to a desired accuracy ($\delta$) as follows

5 $$\begin{aligned} \underset{x,r}{\min }~\Vert e^{-i\textbf{V}(\hat{x})t} - g(x,r) \Vert \le \delta , \end{aligned}$$

where *g*(*x*, *r*) is a polynomial in *x* of degree *r*. To perfectly encode a function of degree *r*, using single and multi-qubit phase gates in a quantum circuit, the total number of gates required is given as follows

6 $$\begin{aligned} N_g=^nC_0 + ^nC_1 + ^nC_2 + \dots + ^nC_r. \end{aligned}$$

In (6), we have an *n* qubit system, and the number of possible multi-qubit phase gates involving *r* qubits will be $^nC_r$ (for e.g., $^nC_1$ - single qubit gates, $^nC_2$ - two-qubit gates, $^nC_3$ - three-qubit gates, and so on). If we are to encode a function of degree *m* perfectly, the quantum system we will need is $n\ge m$ qubits. If $n=m$, the number of gates we will have in that case will be $N_g=^nC_0 + ^nC_1 + ^nC_2 + \dots + ^nC_n = 2^n$. The Appendix has the proof of the above statements.

The gate requirements agree with the encoding strategy described by Grover in^30^ for creating integrable probability distributions. The constant term corresponding to $^nC_0$ can be encoded in the global phase using this procedure. The linear coefficients corresponding to the $^nC_1$ terms require *n* number of single-qubit gates, and the quadratic coefficients corresponding to the $^nC_2$ terms require two-qubit entangled gates of $^nC_2$ combinations, and so on till $^nC_r$ for $r^{th}$-order polynomial fitting. However, to reduce the complexity of the circuit, we can approximate the potential curve to a second or third-order polynomial and can choose single and two-qubit gates (and 3 qubit entangled gates if required) to design a quantum circuit which can reconstruct the potential curve to a desired accuracy as given in (5). The gate parameters may be estimated to do any of the two following tasks.

1. Perfectly encode $K=^nC_0+ ^nC_1+ ^nC_2$ functional values with *K* parameters and not worry about the remaining $2^n - K$ functional values.
2. Give a least squares solution for all the $2^n$ functional values with the above *K* parameters, albeit with some errors.

We take the example of a least squares solution in a 3 qubit quantum circuit, where a potential operator can be constructed. By placing $\theta _0$ as the global phase, phase gates ($P(\theta )$) with angles $\theta _1,\theta _2,\theta _3$ (respectively on the first, second and third qubits) and 2-qubit controlled phase gates ($Cp(\theta )$) with phases $\theta _{12},\theta _{13},\theta _{23}$ (between qubit first and second, first and third, and second and third qubits, respectively) in the quantum registers, we will get a unitary operation that corresponds to a diagonal matrix $\tilde{\textbf{U}}(\theta ) \in \mathcal {C}^{8\times 8}$. $\tilde{\textbf{U}}(\theta )$ is an approximation of the matrix exponential $e^{-i\textbf{V}(\hat{x})t}$, with the matrix $\textbf{V}(\hat{x})$ being a diagonal matrix with elements $\textbf{v}=[v_0,\dots , v_7]$ in its diagonal. Here, the first qubit refers to the lsqb, and the third qubit refers to the msqb.

We have already seen in Fig. 1b and in Fig. 1a that the diagonal elements correspond to a particular state (lattice point); $v_0$ corresponds to the state $\mathinner {|{000}\rangle }$, $v_1$ to $\mathinner {|{001}\rangle }$, etc. Let us now consider the following points.

- Every time we add a phase gate, $P(\theta )$, to a qubit, all states that have that qubit in state $\mathinner {|{1}\rangle }$ get affected by an imaginary exponential of that phase parameter. For example, the phase gate parameter $\theta _1$ will affect all the states where the first qubit (lsqb) is in state $\mathinner {|{1}\rangle }$ - $e^{i\theta _1}\mathinner {|{001}\rangle }, e^{i\theta _1}\mathinner {|{011}\rangle }, e^{i\theta _1}\mathinner {|{101}\rangle }$ and $e^{i\theta _1}\mathinner {|{111}\rangle }$.
- Every time we add a controlled phase gate or a two-qubit phase gate, $Cp(\theta )$, all states that have those qubits in state $\mathinner {|{1}\rangle }$ get affected. For example, the phase gate $\theta _{12}$ will affect all the states where the first qubit and the second qubit are in state $\mathinner {|{1}\rangle }$ - $e^{i\theta _{12}}\mathinner {|{011}\rangle }$ and $e^{i\theta _{12}}\mathinner {|{111}\rangle }$.

Putting all the $P(\theta )$ and $Cp(\theta )$ gates together along with the global phase $\theta _0$, if we compare the exponential in the diagonal matrix $\tilde{\textbf{U}}(\theta )$ and the matrix exponential $e^{-i\textbf{V}(\hat{x})} (t=1)$, we get the following equations in matrix form with *K* ($^3C_0+ ^3C_1+ ^3C_2 = 7$) parameters as

7 $$\begin{aligned} \begin{bmatrix} 1&{} 0&{} 0&{} 0&{} 0&{} 0&{} 0\\ 1&{} 1&{} 0&{} 0&{} 0&{} 0&{} 0\\ 1&{} 0&{} 1&{} 0&{} 0&{} 0&{} 0\\ 1&{} 1&{} 1&{} 0&{} 1&{} 0&{} 0\\ 1&{} 0&{} 0&{} 1&{} 0&{} 0&{} 0\\ 1&{} 1&{} 0&{} 1&{} 0&{} 1&{} 0\\ 1&{} 0&{} 1&{} 1&{} 0&{} 0&{} 1\\ 1&{} 1&{} 1&{} 1&{} 1&{} 1&{} 1 \end{bmatrix}\begin{bmatrix} \theta _0\\ \theta _1\\ \theta _2 \\ \theta _3 \\ \theta _{12} \\ \theta _{13} \\ \theta _{23} \end{bmatrix} = \begin{bmatrix} v_0\\ v_1\\ v_2 \\ v_3 \\ v_4 \\ v_5 \\ v_6 \\ v_7 \end{bmatrix}. \end{aligned}$$

Equation (7) has the form $\textbf{A} \varvec{\xi } = \textbf{v}$, with $\textbf{A}\in \mathbb {R}^{8\times 7}$ being the matrix, $\varvec{\xi }\in \mathbb {R}^{7\times 1}$ denotes the parameter vector, and $\textbf{v}\in \mathbb {R}^{8\times 1}$ represents the vector with functional values corresponding to the potential energy curve. More generally, with a finite number of *n* qubits, the dimension of $\textbf{A}$ will be $N\times K$ with $\textbf{v}\in \mathbb {R}^{N}$ and $K=^nC_0+ ^nC_1 + ^nC_2+\dots + ^nC_r$ parameters (for *r*th order polynomial approximation). One can perform a least square estimate of the parameter vector $\varvec{\xi }$ as

8 $$\begin{aligned} \underset{\theta }{\min }\ ~\Vert \textbf{A}\varvec{\xi } - \textbf{v} \Vert _2, \end{aligned}$$

where $\varvec{\xi } \in \mathbb {R}^K$. The least square solution for (8) can obtained as $\hat{\varvec{\xi }}=\textbf{A}^{+} \textbf{v}$, where $\textbf{A}^{+}$ is the Moore-Penrose inverse of $\textbf{A}$. Note that, while solving for the least square problem, one can rearrange the rows of the matrix $\textbf{A}$ to make it a lower triangular matrix. For example, (7) can be written in the lower-triangular form as follows

9 $$\begin{aligned} \begin{bmatrix} 1&{} 0&{} 0&{} 0&{} 0&{} 0&{} 0\\ 1&{} 1&{} 0&{} 0&{} 0&{} 0&{} 0\\ 1&{} 0&{} 1&{} 0&{} 0&{} 0&{} 0\\ 1&{} 0&{} 0&{} 1&{} 0&{} 0&{} 0\\ 1&{} 1&{} 1&{} 0&{} 1&{} 0&{} 0\\ 1&{} 1&{} 0&{} 1&{} 0&{} 1&{} 0\\ 1&{} 0&{} 1&{} 1&{} 0&{} 0&{} 1\\ 1&{} 1&{} 1&{} 1&{} 1&{} 1&{} 1 \end{bmatrix}\begin{bmatrix} \theta _0\\ \theta _1\\ \theta _2 \\ \theta _3 \\ \theta _{12} \\ \theta _{13} \\ \theta _{23} \end{bmatrix} = \begin{bmatrix} v_0\\ v_1\\ v_2 \\ v_4 \\ v_3 \\ v_5 \\ v_6 \\ v_7 \end{bmatrix}. \end{aligned}$$

This operation efficiently estimates the parameters ($\theta _0,~\dots ,~\theta _{23}$) a priori for the quantum encoding. This is a one-time operation and can be considered as a pre-processing step.

**Figure 2:**
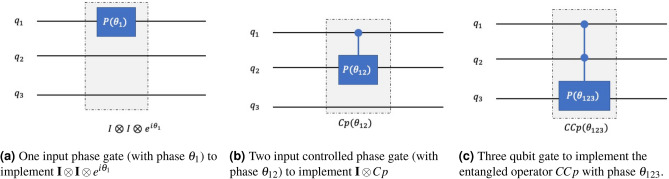
Circuit construction with polynomial approximation encoding in a 3 qubit circuit: Here, the input qubits are denoted by $q_1,q_2$, and $q_3$.

With polynomial approximation encoding, we seek to implement quantum circuits that closely approximate (up to a finite order) the time evolution operation. We select the phase gates to prepare the diagonal matrix. Here, 1-qubit phase gates can approximate the time evolution operation to the first order. Similarly, two-qubit phase gates (*Cp*) and three-qubit phase gates (*CCp*) can be used to approximate up to 2nd, and 3rd order polynomial. In Fig. 2, we have shown the circuit implementation with different gates (1-input, 2-input, and 3-input gates) to prepare a diagonal matrix with desired phase angles. The phase and entangled phase gates used in this figure are defined as follows

10 $$\begin{aligned} P(\theta _1)&=\begin{bmatrix} 1&{}0\\ 0&{}e^{i\theta _1} \end{bmatrix},\end{aligned}$$

11 $$\begin{aligned} Cp(\theta _{12})&= \textbf{I}\otimes \mathinner {|{0}\rangle }\mathinner {\langle {0}|}~ + ~P(\theta _{12})\otimes \mathinner {|{1}\rangle }\mathinner {\langle {1}|}\nonumber \\&= \begin{bmatrix} 1&{}0&{}0&{}0\\ 0&{}1&{}0&{}0\\ 0&{}0&{}1&{}0\\ 0&{}0&{}0&{}e^{i\theta _{12}} \end{bmatrix},\end{aligned}$$

12 $$\begin{aligned} CCp(\theta _{123})&= \textbf{I}\otimes \textbf{I}\otimes \mathinner {|{0}\rangle }\mathinner {\langle {0}|}~ + ~Cp(\theta _{123})\otimes \mathinner {|{1}\rangle }\mathinner {\langle {1}|}\nonumber \\&=\begin{bmatrix} 1&{}0&{}0&{}0&{}0&{}0&{}0&{}0\\ 0&{}1&{}0&{}0&{}0&{}0&{}0&{}0\\ 0&{}0&{}1&{}0&{}0&{}0&{}0&{}0\\ 0&{}0&{}0&{}1&{}0&{}0&{}0&{}0\\ 0&{}0&{}0&{}0&{}1&{}0&{}0&{}0\\ 0&{}0&{}0&{}0&{}0&{}1&{}0&{}0\\ 0&{}0&{}0&{}0&{}0&{}0&{}1&{}0\\ 0&{}0&{}0&{}0&{}0&{}0&{}0&{}e^{\theta _{123}} \end{bmatrix}. \end{aligned}$$

As an example, we have shown that placing a phase gate with angle $\theta _1$ can be used to implement an operator $\textbf{I}\otimes \textbf{I} \otimes e^{i\theta _1}$ for a 3 qubit quantum circuit shown in Fig. 2a. The unitary operator obtained by placing a phase gate ($P(\theta _1)$) in the first qubit is given by

13 $$\begin{aligned} \textbf{U}_1 = \begin{bmatrix} 1&{}0&{}0&{}0&{}0&{}0&{}0&{}0\\ 0&{}e^{i\theta _1}&{}0&{}0&{}0&{}0&{}0&{}0\\ 0&{}0&{}1&{}0&{}0&{}0&{}0&{}0\\ 0&{}0&{}0&{}e^{i\theta _1}&{}0&{}0&{}0&{}0\\ 0&{}0&{}0&{}0&{}1&{}0&{}0&{}0\\ 0&{}0&{}0&{}0&{}0&{}e^{i\theta _1}&{}0&{}0\\ 0&{}0&{}0&{}0&{}0&{}0&{}1&{}0\\ 0&{}0&{}0&{}0&{}0&{}0&{}0&{}e^{i\theta _1} \end{bmatrix}. \end{aligned}$$

The placing of this unitary operator will lead to phase operations $e^{i\theta _1}\mathinner {|{001}\rangle }, e^{i\theta _1}\mathinner {|{011}\rangle }, e^{i\theta _1}\mathinner {|{101}\rangle }$ and $e^{i\theta _1}\mathinner {|{111}\rangle }$, as explained in the previous section. Similarly, one can get the operator $\textbf{I}\otimes P(\theta _2)\otimes \textbf{I}$ by placing a phase gate $P(\theta _2)$ with angle $\theta _2$ in the second register, and so on till the last qubit. We can place *n* phase gates to an *n*-qubit circuit to approximate a first-order polynomial for the potential energy function.

For the second-order approximation, we need 2-qubit quantum gates. They are the controlled phase gates and are denoted by *Cp*. For a *n* qubit quantum system, there are $^nC_2$ possible combinations to place different *Cp* gates in the circuit. Similarly, we can use a *CCp* gate to prepare a unitary of the form given in (12). By placing phase gates (with angles $\theta _1, \theta _2, \theta _3$) and controlled phase gates (with angles $\theta _{12},\theta _{13},\theta _{23}$), we can get a linear combination of angles in the diagonal of the unitary matrix as shown in (7). Finally, by solving the linear equations in the least square sense, we obtain the estimated angles (parameters) to prepare the polynomial approximation of the time evolution operation.

## Results

**Figure 3:**
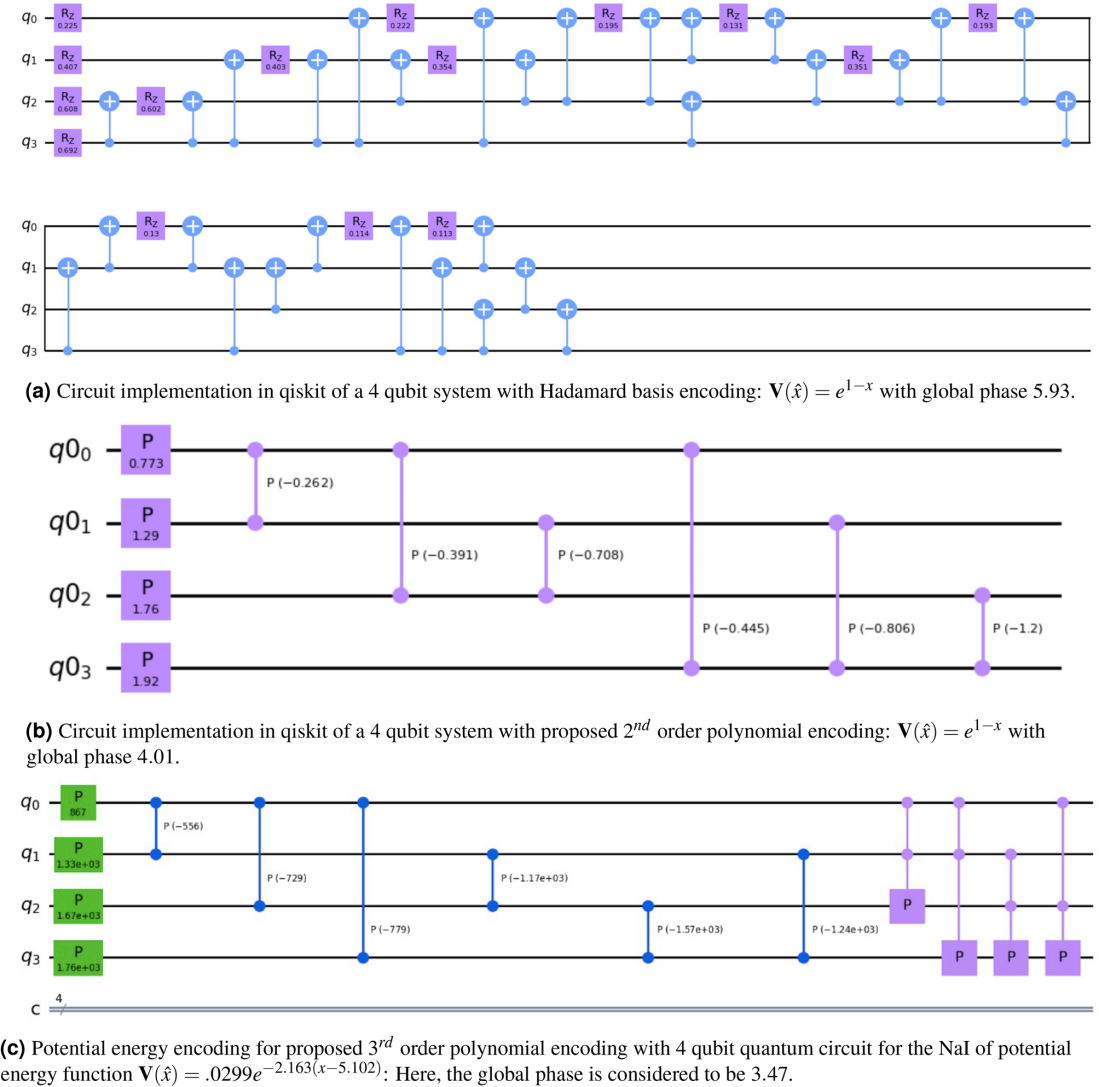
Quantum encoding of the potential energy operator using 4 qubit quantum circuits: In ($\textbf{a}$) Hadamard encoding circuit takes 15 Rz operator and 34 CNOT gates, Proposed polynomial encoding with 2nd order approximation in ($\textbf{b}$) we need 4 phase gates, and 6 Cp gates, and for 3rd order approximation shown in ($\textbf{c}$) the proposed encoding takes 4 phase gates, 6 phase gates, and 4 CCp gates. It shows the difference between circuit depth and use of computational resources for the energy function encoding on a quantum computer by standard Hadamard basis encoding versus the proposed polynomial encoding.

This section demonstrates numerical simulations for the time evolution operator design and potential energy reconstruction performed on IBM quantum simulators using qiskit^24,31^. In the below subsections, we show the implementation of the potential energy operator for 3 and 4 qubit systems as examples, using the proposed Hadamard basis encoding and polynomial approximate encoding methods. The performance of the proposed framework of the time evolution operator is measured with gate complexity as the key parameter index (KPI). In Table 2, we have shown the parameters taken in the simulation environment. Kindly note that the unit of the potential energy in this study is Hartree, and the distance coordinate is taken in Å  in the numerical results.

**Table 2:** Simulation parameters.

Quantum simulator	Unitary simulator, Qasm simulator
IBMQ machine	ibmq_mumbai, ibm_nairobi^32^
Number of qubits (*n*)	3–10
Number of shots	10, 000
Range of space coordinate (*x*)	[0, 10] (Å)
Sampling interval (*dx*)	$10/{2^n}$
Potential energy curve	$V(x)= a_1e^{-a_2(x-r_1)}$
Example	Potential energy of NaI

### Encoding potential energy with 4 qubit quantum circuit using Hadamard basis encoding and 2nd order polynomial approximation

Figure 3a gives the quantum circuit for a 4 qubit system as implemented in qiskit. With a 4 qubit system, the potential energy function $\textbf{V}(\hat{x})\in \mathbb {R}^{16\times 1}$ is mapped to the computational bases $\mathinner {|{0000}\rangle }$ to $\mathinner {|{1111}\rangle }$. The term with the basis corresponding to $\textbf{I}^{\otimes 4}$ is encoded as the global phase. The number of basis states prepared with the combination of Pauli-*Z* operators is $2^4=16$.

**Figure 4:**
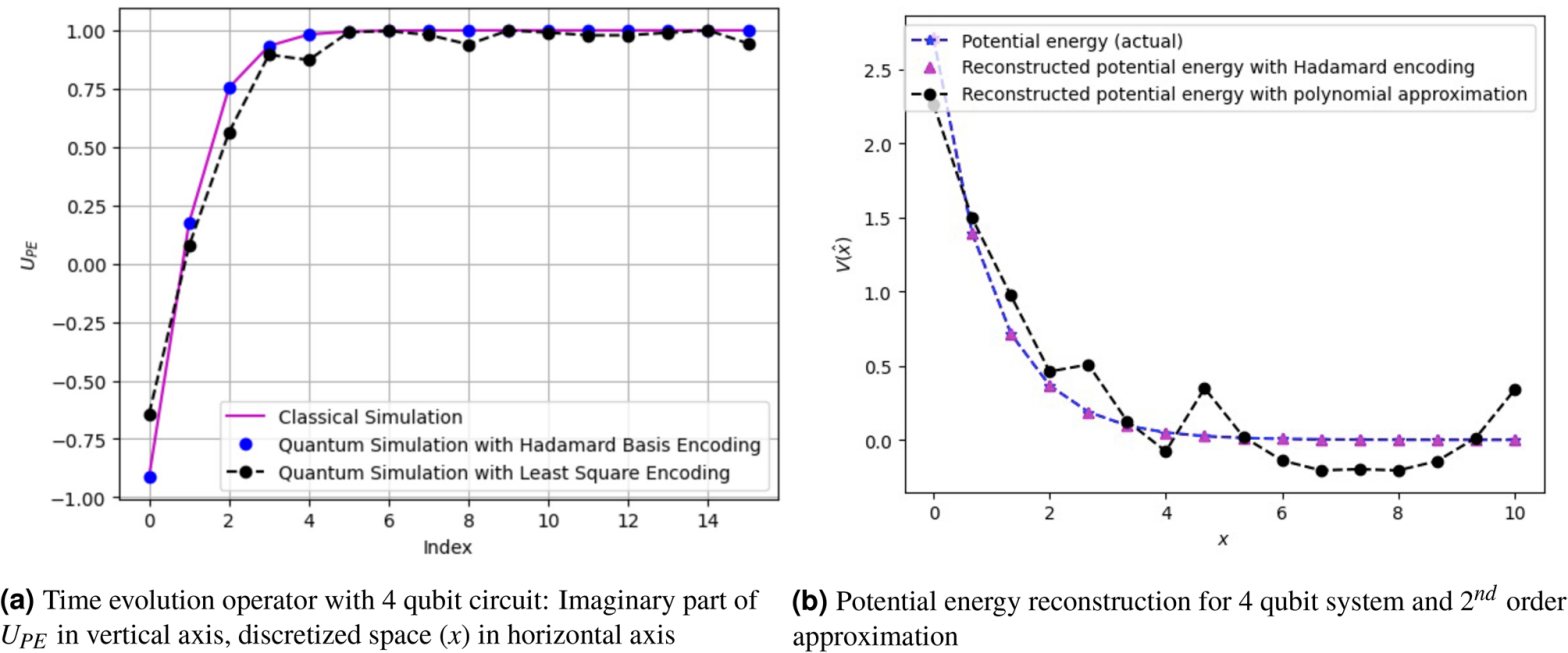
Reconstruction of potential energy operator using a 2nd order polynomial encoding approximation and Hadamard encoding: In (**a**), the imaginary part of the time evolution operation $e^{-i\textbf{V}t}$ is plotted using the reconstructed $\textbf{V}$, and compared with a classical simulation. As expected Hadamard basis encoding is a perfect match. It can be seen that the 2nd order approximation is close to the classical results with some errors. In (**b**) the plots of the reconstructed potential energy function $\textbf{V}(\hat{x})=e^{1-x}$ are shown. Here, the unit of distance is in Å, and energy in Hartree.

To encode with Hadamard basis, we used the combination of *Rz* and CNOT gates. For encoding in Hadamard basis for 4-qubits, one would need 15 number of single-qubit gates (*Rz*), and 34 two-qubit gates (CNOT). However, on further optimization, the number of CNOT gates can be reduced^33^. Fig. 3 gives a circuit which requires 30 CNOT gates. The circuit we have provided here is a generic circuit upon which further circuit optimizations are possible, as shown in Ref^23^. The number of CNOT gates can be considered an upper bound on the number of two-qubit gates required. The quantum Hadamard encoded unitary matrix $\textbf{U}_{PE}$ approximates the function arbitrarily close to the classical function. We have performed a quantum simulation with evolution time $\Delta t=1$, and potential energy function $\textbf{V}(\hat{x})=e^{1-x}$ on qiskit unitary simulator, and the corresponding plot of the diagonal vector is given in Fig. 4.

In Fig. 3b, we show a 4-qubit quantum circuit with the 2nd-order approximate polynomial encoding. Here, we have used four 1-qubit phase gates ($^4C_1$) and six Cp gates ($^4C_2$). Hence, the total number of quantum gates used is 10. Here, the time evolution operator (corresponding to the potential energy), i.e., $e^{-i\textbf{V}(\hat{x})t}$ is approximated with 2nd-order polynomial approximation function *g*(*x*, 2) with 10 parameters (phases of the quantum gates). On including the global phase, we have a total of 11 parameters for the least square estimate of the parameter vector $\hat{\varvec{\xi }}$.

An exponentially decaying potential energy function of the form $\textbf{V}(\hat{x})=e^{(1-x)}$ is simulated with Hadamard basis encoding and polynomial approximation encoding method as shown in Fig. 4. In Fig. 4a, the plot of the diagonal unitary operator $U_{PE}=e^{-i\textbf{V}t}$ is given (imaginary values of the plot are shown here). The potential energy is discretized to 16 samples ($v_0,\dots , v_{15}$). The Hadamard basis encoding method constructs the unitary operator arbitrarily close to the classically encoded values ($e^{-iv_0 t}, \dots , e^{iv_{15}t}$). The approximate encoding with the least squares method (for 2nd order polynomial approximation of the potential energy function) gives an approximation of the classically encoded potential energy function.

In Fig. 4b, we have shown the reconstruction plot of the potential energy function ($V(\hat{x})$) obtained from the simulated unitary operator. The signature of the reconstruction is close to the original. The reconstruction of the potential energy function using the least square encoding is slightly degraded as compared to the Hadamard-based encoding. However, one can simulate the polynomial approximation on a real quantum computer with fewer computational resources. For example, for a 4 qubit quantum circuit in a simulator, we needed 10 single and two-qubit gates for the 2nd order polynomial approximation encoding. In contrast, the Hadamard-based encoding-based circuit requires 49 quantum gates. *Note:* The gate count of 10 for the 2nd order polynomial basis encoding and the gate count of 49 in the Hadamard basis encoding are the pre-transpilation gate counts in a simulator. On transpilation (ibmq_mumbai), both methods have different gate counts, as shown in Table 3.

**Table 3:** Gate counts before (simulator) and after transpilation (ibmq_mumbai).

Encoding		Gate counts				
		CNOT	RZ	SX	X	Total
Pre-transpilation	Hadamard basis	34	15	–	–	49
	Proposed polynomial	6	4	–	–	10
Post-transpilation	Hadamard basis	47	32	12	2	93
	Proposed polynomial	18	18	–	–	36

###  Potential energy evolution with 3rd-order polynomial approximation using a 3-qubit quantum system

**Figure 5:**
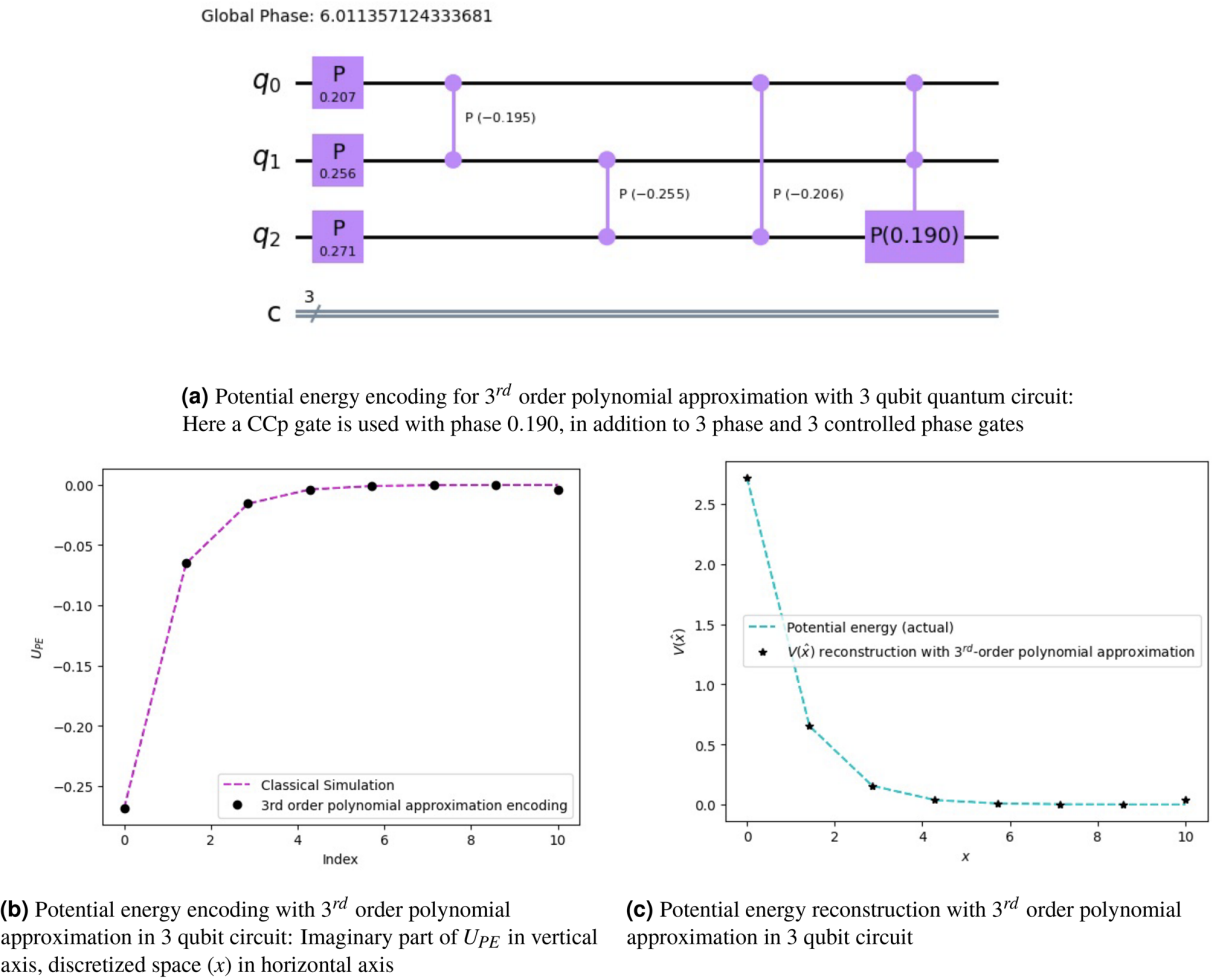
Potential energy encoding and reconstruction using polynomial encoding with 3rd approximation in 3-qubit quantum circuit: (**a**) shows the quantum circuit representation with 3 phase gates, 3 Cp gates, and one CCp gate, (**b**) gives the imaginary part of the time evolution operation with the circuit in (**a**,**c**) gives the reconstructed potential energy function. By increasing the order of the approximation the reconstruction has become more accurate as compared to Fig. 4. A total of $2^3 = 8$ parameters are required; 7 gate parameters and one for the global phase.

One can take a higher-order polynomial approximation to improve the performance of the least squares encoding of the potential energy. For example, 3-qubit quantum gates (e.g., controlled-controlled phase (CCp) gate) can be used in addition to phase and controlled phase gates (Cp) for a 3rd order polynomial approximation of the potential energy. An example is shown in Fig. 5a for a 3-qubit quantum circuit. We have used three phase gates ($^3C_1$), three Cp gates ($^3C_2$), and one CCp gate ($^3C_3$). Consequently, we have 8 parameters (including the 1 global phase parameter) to be estimated from the least squares formulation. The CCp gate may be constructed as detailed on the page 182 of Ref^34^. The construction of the evolution operator $U_{PE}$, upon using this 3rd order polynomial approximation, is shown in Fig. 5b, and the reconstruction of the potential energy $V(\hat{x})$ is shown in Fig. 5c. This shows that with additional higher-input quantum gates, the approximation accuracy can be improved.

### Example: time evolution due to potential energy function in NaI

A prime example often chosen in the femtochemistry literature^35^ to study the wave packet motion in the presence of a potential is the Sodium Iodide (NaI) molecule. The analytical expression of the potential energy ($V(\hat{x})$) of NaI as a function of atomic distance (*x*) is given by

14 $$\begin{aligned} V(x)= a_1 e^{-a_2 (x-r_1)}, \end{aligned}$$

with $a_1=0.0299$, $a_2=2.163$, and $r_1=5.102$ respectively.

#### Reconstruction with 2nd order polynomial

**Figure 6:**
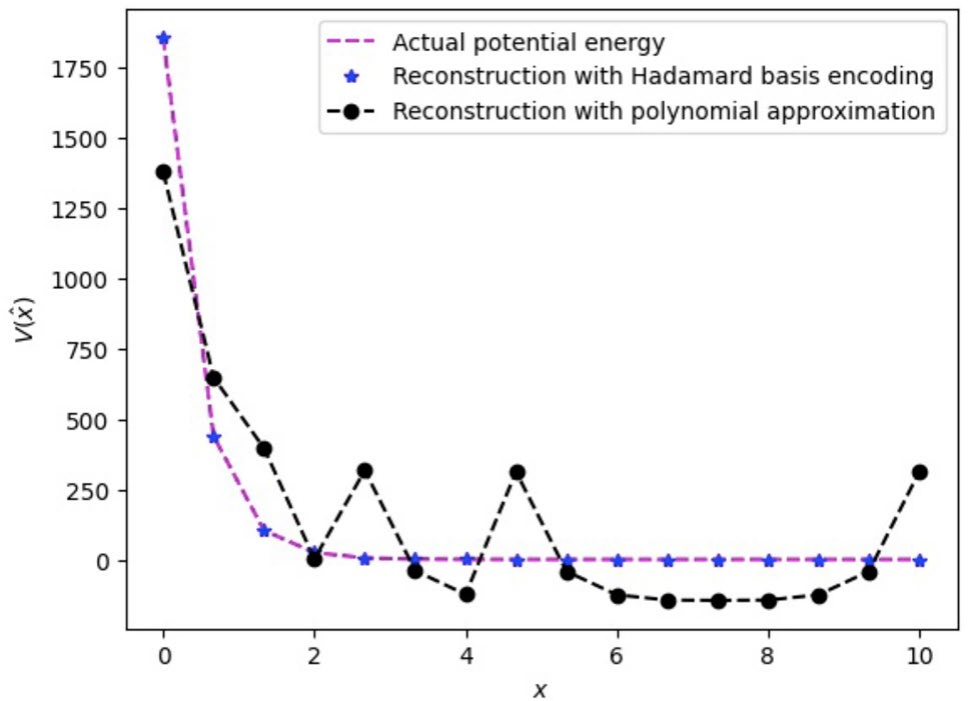
Potential energy reconstruction with Hadamard basis encoding and polynomial encoding with 2nd order approximation: This is constructed with the circuit given in Fig. 1b with a different set of parameters obtained for NaI with the functional form $\textbf{V}(\hat{x})=0.0299 ~e^{-2.163(x-5.102)}$. Here, we have a total 11 parameters (10 gate parameters and 1 global phase). With $2^4 = 16$ unknown variables, the approximation is justifiably deviated from the actual curve.

Figure 6 shows the reconstruction of the potential energy function with the two proposed techniques, i.e., Hadamard basis encoding and the polynomial approximation encoding. Here, we have used 4 qubits to encode potential energy functions on a quantum computer. The Hadamard basis encoding circuit reconstructs the potential energy with high closeness to the actual (classical) potential energy curve. We have already seen that a second-order polynomial approximation with 10 quantum gives us an approximation to the actual potential energy function. The quantum circuit for the 2nd order approximation of polynomial encoding is similar to Figure 3b with a different set of parameters. It is important to note here that, although Hadamard basis encoding, theoretically, gives a very close approximation to the actual potential energy, it may suffer from poor fidelity as compared to the polynomial approximation method in a real quantum computer.

#### Reconstruction with 3rd order polynomial

**Figure 7:**
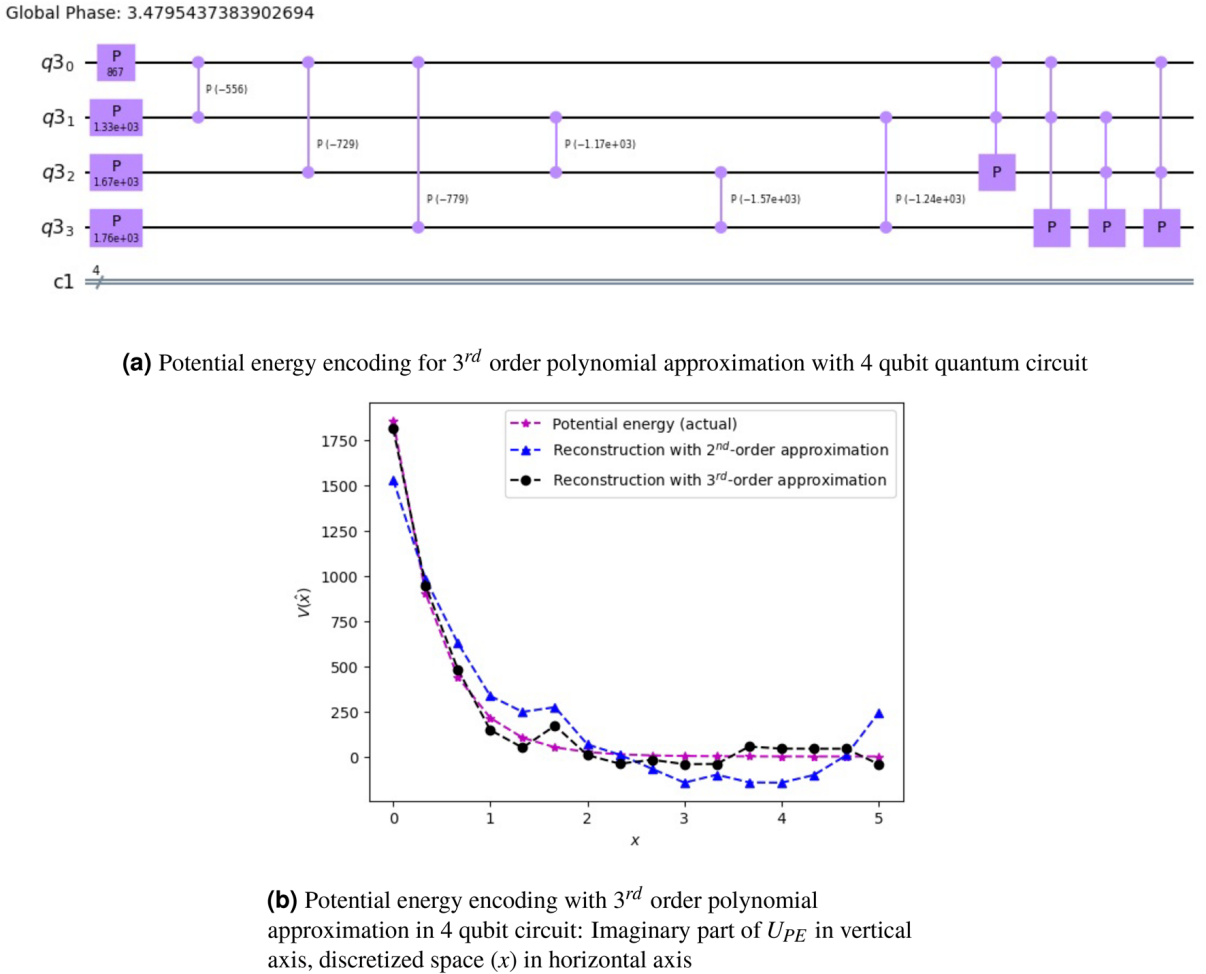
Reconstruction of potential energy function of NaI using 3rd polynomial approximation on a 4-qubit quantum circuit: (**a**) denotes the circuit representation with 15 parameters (including 14 gate parameters, and one global phase), and (**b**) represents the reconstructed potential energy curve. By increasing the order of approximation here, we have got closer to the actual curve, as opposed to the 2nd order approximation in Fig. 6.

The potential energy reconstruction with a 4 qubit quantum circuit for the 3rd approximation is shown in Fig. 7. The quantum circuit as shown in Fig. 7a consists of 4 phase and 6 Cp gates (same as the 2nd order approximation). In addition, we have added another 4 CCp gates ($^4C_3$). The performance of the $3^{rd}$ order approximation as compared to the 2nd order approximation is shown in Fig. 7b. It is observed that increasing the order results in better reconstruction at the cost of additional resources. Note that the above reconstruction is based on simulator results. The hardware performance is given in the next section.

### Fidelity performance

The evolution due to a complex diagonal matrix preserves the squared amplitude or the probability distribution of the wave function since $\vert ae^{i\theta } \vert ^2 = \vert a \vert ^2$. As such, the fidelity of the evolved state with respect to the initial state should ideally be 1.0. However, because of decoherence, the fidelity goes down with circuit depth. In order to measure the effectiveness of these algorithms in present-day quantum hardware and to see the impact the circuit depth may have on the fidelity, we used a swap test to measure the fidelity.

**Table 4:** Fidelity performance for 3 qubit system on Qasm simulator and ibm_nairobi^32^.

Hamiltonian encoding	Qasm simulator	Hardware (ibm_nairobi)	FakeNairobi
Hadamard encoding	1.0	0.28	0.59
2nd order poly encoding	1.0	0.46	0.68
3rd order poly encoding	1.0	0.40	0.63

**Table 5:** Fidelity performance for 4 qubit system on Qasm simulator and ibm_mumbai^32^.

Hamiltonian encoding	Qasm simulator	Hardware (ibmq_mumbai)	FakeMumbai
Hadamard encoding	1.0	0.18	0.51
2nd order poly encoding	1.0	0.33	0.7
3rd order poly encoding (1 CCp)	1.0	0.27	0.63
3rd order poly encoding (2 CCp)	1.0	0.2	0.62
3rd order poly encoding (3 CCp)	1.0	0.12	0.61
3rd order poly encoding (4 CCp)	1.0	0.09	0.57

We initialised the reference circuit with all qubits at state at $\mathinner {|{0}\rangle }$ for measuring the fidelity. Table 4 gives the fidelity performance for a 3 qubit circuit. In a Qasm simulator, as expected, every method performs equally well, and we get perfect fidelity. For the 3 qubit circuit, we ran our experiments on an IBM quantum machine called ibm_nairobi. The proposed 2nd order approximate polynomial encoding method is observed to outperform Hadamard encoding and higher-order approximation with a fidelity of 0.46.

We experimented similarly for a 4 qubit circuit on another IBM machine called ibmq_mumbai. The 4 qubit circuit, with a larger depth than the 3 qubit circuit, sees a larger drop in fidelity. Table 5 gives the details. The Hadamard encoding method achieves a fidelity of 0.18, whereas the 2nd order polynomial encoding has an improved fidelity of 0.33. We have also shown the fidelity of 3rd order polynomial approximation encoding.

Note: We also ran our experiments on fake backend machines (FakeNairobi in Table 4, and FakeMumbai in Table 5) to consider noise characteristics in the simulations. These Fake machines show that the polynomial encoding scheme is robust compared to the Hadamard basis encoding. For example, in the 3 qubit circuit, 2nd order polynomial encoding achieves a fidelity of 0.68, and a quantum circuit with 3rd order polynomial encoding with 1 CCp gate has a fidelity of 0.63.

The above experiments on real quantum hardware show a comparative study of the proposed quantum encoding methods for encoding the potential energy function. Clearly, there are trade-offs we need to consider when going for more accurate algorithms in hardware.

### Gate complexity

We will discuss a quantitative picture of the computational resources required by the two encoding schemes, viz. Hadamard basis encoding and polynomial approximation encoding. The two-qubit gate complexity with Hadamard basis encoding is given in Eq. (4). The two-qubit gate complexity with 2nd order polynomial basis encoding is given in Lemma 1. As the system size (number of qubits) increases, the gap in gate complexity between the proposed polynomial encoding and that of the Hadamard basis encoding also increases.

It is observed that the computational resource required by the polynomial approximate encoding is much less than the Hadamard encoding for a higher number of qubits. However, the Hadamard basis encoding method has higher construction accuracy (of the unitary time evolution operator) and reconstruction (of the potential energy function), as it uses $2^n$ bases to encode $2^n$ functional points. The polynomial approximate method is a trade-off between accuracy and complexity. For practical quantum encoding of the time evolution operation on a real quantum machine, polynomial approximate encoding could be beneficial, as it uses fewer computational resources with improved fidelity, as compared to Hadamard-based encoding.

#### Lemma 1

For encoding a potential energy function up to $r^{th}$ order polynomial approximation, we need $\tilde{\mathcal {O}}\left( \sum _{j=2}^{r} ~^nC_j(2^{j}-3)\right)$ number of 2-qubit gates.

#### Proof

To encode a *r*th order polynomial approximation, we will need $^nC_1$ single qubit gates, $^nC_2$ two-qubit gates, and so on till $^nC_r$ *r*-qubit gates. A *j*-qubit gate has $j-1$ control qubits, i.e., $C^{j-1}p$ phase gate. Implementing a $C^{j-1}p$ phase gate in a quantum computer requires $(2^{j-1}-2)$ CNOT gates, and $2^{j-1} - 1$ number of *Cp* gates. Overall, we will need $(2^{j}-3)$ two qubit gates^34^. If we add up all the multi-qubit gates, starting from $j=2$ to $j=r$, we will get the expression given in the above lemma. Single qubit gates are ignored as they are inexpensive. This can be further optimized using circuit optimization tools in qiskit. $\square$

The detailed encoding technique with the proposed approximate polynomial method is given in the “Appendix”.

**Table 6:** Upper bound on the number of two-qubit gate counts of Hadamard basis encoding and proposed polynomial basis encoding (2nd order to 5th order approximation).

Qubits	Hadamard encoding	2nd order approx	3rd order approx	4th order approx	5th order approx
3	10	3	8	–	–
4	34	6	26	39	–
5	98	10	60	125	154
6	258	15	115	310	484
7	642	21	196	651	1260
8	1538	28	308	1218	2842
9	3586	36	456	2094	5748
10	8194	45	645	3375	10,683
11	18,434	55	880	5170	18,568
12	40,962	66	1166	7601	30,569
13	90,114	78	1508	10,803	48,126
14	196,610	91	1911	14,924	72,982
15	425,986	105	2380	20,125	107,212
16	917,506	120	2920	26,580	153,252
17	1,966,082	136	3536	34,476	213,928
18	4,194,306	153	4233	44,013	292,485
19	8,912,898	171	5016	55,404	392,616
20	18,874,370	190	5890	68,875	518,491

We have shown a comparison table of two-qubit gate counts for the proposed polynomial basis encoding and the Hadamard basis encoding method in Table 6. It shows that 2nd, and 3rd order approximation in the proposed polynomial basis encoding require a lesser number of two-qubit quantum gates as compared to Hadamard basis encoding for all systems. In the case of 4-order approximation, we can expect an advantage in the two-qubit gate counts when the encoding is done on an 8 qubit (or more) quantum system. It also follows that for the 5 order approximation, an advantage is expected when the system is of 12 qubits or more. In general, a significant gate count advantage may be achieved when the encoding is done in higher qubit systems.

It is to be noted here that the two-qubit gate counts given in Table 6 are based on an algorithmic analysis. During implementation on real quantum hardware, these numbers will change based on qubit connectivity. For an all-to-all qubit connectivity, the numbers will remain the same. However, the numbers will increase for a different processor topology (heavy-hex lattice, a rectangular array, etc.) due to the requirement of multiple swap operations to implement long-range two-qubit gates. This is true for both Hadamard encoding and approximate polynomial encoding.

### Analysis of error

Let, $\textbf{U}_{f}$ denote the unitary diagonal matrix with the vector $[e^{i f_0 \Delta t},\dots , e^{i f_{N-1} \Delta t}]$ (containing the samples of the function *f*(*x*) evolved for the duration $\Delta t$) in its diagonal. Let $\textbf{U}_{p}$ be the unitary diagonal matrix (approximating *f*(*x*) up to polynomial order *r*) that the quantum circuit represents. We approximate the polynomial encoding of a diagonal Hamiltonian, with its elements sampled from *f*(*x*), with a suitable polynomial function *p*(*x*) of order *r*, such that

15 $$\begin{aligned} \Vert \textbf{U}_{f} - \textbf{U}_{p} \Vert _2 \le \epsilon _b \end{aligned}$$

is bounded within the absolute error $\vert \epsilon _b \vert \ge 0$. Considering an evolution time of $\Delta t$, the Hamiltonian simulation problem in (15) can be rewritten as

16 $$\begin{aligned} \Vert e^{-i \textbf{F} \Delta t}- \textbf{U}_{p} \Vert _2 \le \epsilon _T + \epsilon _g + \epsilon _d + \epsilon _{cr}, \end{aligned}$$

where $\textbf{F}$ is a diagonal matrix with the vector (formed by samples of the function *f*(*x*)) in its principal diagonal, $\epsilon _T\ge 0$ denotes the truncation error, and $\epsilon _g\ge 0$ is the gate-level error incurred in simulating $\textbf{U}_p$ on a real quantum machine. The term $\epsilon _d$ denotes the error due to an error in coherence time, and $\epsilon _{cr}$ represents the cross-talk and readout error.

The intuition behind the consideration of $\textbf{U}_{p}$ for the Hamiltonian simulation problem is that $\textbf{U}_{p}$ has a quantum representation easier than $\textbf{U}_{f}$ in computational gate complexity sense. The algorithm with polynomial encoding will require $\mathcal {O}(^nC_r, 1/{\epsilon _b}, 1/{\epsilon _g})$ gates approximately (with $\epsilon _b=\epsilon _T + \epsilon _d + \epsilon _{cr}$) and no ancilla qubits.

#### Truncation error

In general, the polynomial expansion of *p*(*x*) up to order *n* can be written as,

17 $$\begin{aligned} p(x)=a_0 + a_1 x + a_2 x^2 + a_3 x^3+ \dots + a_n x^n. \end{aligned}$$

On truncating the function *p*(*x*) up to order *r*, the truncation error becomes $\mathcal {O}(h^{r+1})$ where *h* is the distance between two successive samples (also called step size). For example, if 10 qubits are taken to encode the function *f*(*x*) within a region [0, 1], the *h* can be approximately equal to $9.7\times 10^{-4}$. With an increase in sample size of the function *p*(*x*) with increased qubit size, the value of *h* can be optimized further. Hence, the truncation error follows $\Vert \epsilon \Vert \approx \mathcal {O}(h^{r+1})$.

#### Gate level error variance

Considering that each quantum gate has an average noise variance of $\sigma _g^2$, the overall gate level error for the overall *L* number of quantum gates can be bounded as $\epsilon _g^2 \approx \mathcal {O}(L\sigma _g^2)$. The error level in the 2 qubit gates is reported to be in the order of $10^{-2}$ to $10^{-3}$, whereas single qubit gates incur negligible errors^36,37^. Hence, the dominant error terms are due to the 2 qubit gates, with an overall gate level error given by $L_2\times 10^{-3}$.

#### Decoherence error

The period during which a qubit preserves its information is referred to as coherence time, while the phenomenon of information loss is termed the decoherence process^38^. There are two types of coherence times, namely $T_1$ and $T_2$. $T_1$ is associated with the amplitude damping channel, representing the process where the high-energy state $|1\rangle$ decays to the low-energy state $|0\rangle$. In the amplitude damping channel, the qubit’s state is preserved with a probability of $p_1(t) = e^{-\frac{t}{T_1}}$, where $t$ is the operational time dependent on the cumulative gate times^37^. $T_2$ pertains to the phase damping channel, signifying the phase change process. In the phase damping channel, the qubit maintains its state with a probability of $p_2(t) = e^{-\frac{t}{T_2}}$. Based on these two time-constant parameters, the coherence time error is given as

18 $$\begin{aligned} \epsilon _{d} = 1 - e^{-\Delta t\left( \frac{1}{T_1} + \frac{1}{T_2}\right) }. \end{aligned}$$

Based on our experiments on the IBM quantum machines, the median and mean time taken for the 2-qubit CNOT gates are given by 440.8  *ns*, and 513 *ns* approximately. For the single qubit phase gates, these times are approximately 35 *ns*. It suffices that the CNOT gates are more prone to decoherence in present noisy intermediate-scale quantum (NISQ) computers. Considering the mean coherence error time approximately in $\mathcal {O}(10^{-7})$, the overall decoherence error for $L_2$ number of 2 qubit gates are given by $\Vert \epsilon _{d} \Vert \approx L_2\times 10^{-7}$. Hence, it is important that the time taken for the overall quantum circuit execution is within the coherence time.

#### Cross talk error and readout error

Besides the truncation and gate level errors (including the decoherence part), there are other uncertainty factors, such as the cross-talk error, readout and coupling errors often seen in practical quantum hardware. In quantum circuits, cross-talk error refers to unwanted interactions between qubits, leading to unintended operations or errors in quantum gates. The readout errors occur when the measurement of a qubit’s state produces incorrect results due to imperfections in the measurement process or environmental factors, and the coupling errors stem from imprecise control of qubit-qubit interactions, causing deviations from the desired quantum gate operations and impacting the overall accuracy of quantum computations. Typically, their order of magnitude is similar, and we have shown the readout error from the $ibm\_auckland$ quantum machine in Table 7. Here, the average readout error seems to be 0.0087 based on the measurement of the first 9 qubits. Total errors incurred by the cross-talk, coupling and readout errors depend on the qubit size *n*.

#### A bound on the simulation error

Given the different sources of errors that arise in the practical quantum hardware and the simulation error of our proposed approach, we provide an overall approximate error bound in the lemma given below.

##### Lemma 2

*The overall approximate error bound in the diagonal unitary encoding of the function* *f*(*x*) *using the proposed polynomial encoding procedure within polynomial order* *r* *encoded in* *n* *qubit registers and evolved for time* $\Delta t$ *has the form given in* (19).

19 $$\begin{aligned} \Vert \epsilon _s \Vert \approx \mathcal {O}\left( h^{r+1}\right) + L_2 \sigma _g^2 + \mathcal {O}\left( 1+ \Delta t \left( \frac{T_1+T_2}{T_1 T_2}\right) \right) + \sigma _{cr}^2. \end{aligned}$$

##### Proof

The overall error bound is computed in an approximate sense. Here, the term $\mathcal {O}(h^{r+1})$ denotes the order of truncation error. As the 2 qubit gates are erroneous in the NISQ computers, the gate level error is dependent on the size of the 2-qubit gates ($L_2$), and the overall gate error can be approximated as $L_2\sigma _g^2$ for individual error variance of $\sigma _g^2$. Taking the first-order approximation of the decoherence error term as shown in (18), we get the error to be in $\mathcal {O}\left( 1+ \Delta t \left( \frac{T_1+T_2}{T_1 T_2}\right) \right)$. Finally, we add the total read-out error variance term $\sigma _{cr}^2$ to the error bound, which depends on the actual quantum hardware. $\square$

**A note on mean squared error** The 2-norm distance between the exact potential operator and the proposed polynomial approximate operator is given in (15). The mean squared error (MSE) in the approximation can be measured with the quantity $\Vert \epsilon _s \Vert ^2 + \Vert \epsilon _m \Vert ^2$, which considers both the simulation errors and measurement uncertainty (with error-variance $\epsilon _m^2$). Within the coherence time of evolution, the simulation error term $\Vert \epsilon _s \Vert ^2$ may get reduced with the higher order approximation of the proposed polynomial encoding. The measurement uncertainty error may be optimized with multiple circuit executions. Overall, it is a trade-off between the accuracy of the encoding and the gate complexity.

### Discussion

By simulating potential energy functions at the quantum level, we gain a deeper understanding of the behaviour of molecules. This knowledge is crucial for chemistry, materials science, and bio-sciences, where precise knowledge of molecular interactions is essential. By leveraging the unique properties of quantum mechanics, such as superposition, interference and entanglement, quantum simulations provide a more accurate representation of molecular behaviour. This allows for more precise predictions and analysis, as shown in recent literature^2,34^.

Assuming the first quantization level of molecular dynamics, which describes the energy distribution as a function of inter-nuclear, electron-electron and electron-nuclear interactions, we investigate a diagonal operator involving the potential energy. Diagonal operators play a crucial role in simplifying the simulation of quantum systems, enabling efficient representation of potential energy surfaces.

However, quantum simulations are computationally expensive, especially for larger and more complex molecules. Accurate potential energy encoding is crucial for meaningful simulations on real quantum machines. Developing reliable methods for representing potential energy surfaces depends on choosing a suitable basis encoding. Hadamard basis encoding method can accurately encode a Hamiltonian operator. However, it requires significantly large quantum resources in the form of circuit elements, e.g., two-qubit gates etc. This becomes hard to implement in present-day quantum machines as noise level builds up with circuit depth. Validating the results of quantum simulations against experimental data is thus a challenge because of the computational gate complexity of encoding algorithms.

We propose a conceptually novel framework with our proposed polynomial basis encoding method to bridge the gap between the accurate simulation of quantum chemistry and the high computational gate complexity (thereby limiting the error bound). It reduces the gate-level complexity of the potential energy simulation and gives the user a tool to trade-off between accuracy and complexity by increasing the order of approximation. We have shown how we can significantly reduce the usage of quantum computing resources for simulation problems involving diagonal Hamiltonian with polynomial encoding. We present case studies for the Sodium Iodide molecule, and the experimental results support the propositions. As reported in this article, the key novelties lie in the complexity reduction and fidelity improvement.

## Conclusion

In this work, we have proposed approximate methods to represent potential energy surfaces in Hamiltonian simulation problems in a quantum computer. As we encode the large number of samples of the energy surface in the Hamiltonian, the standard Hadamard basis encoding grows by $2^{n}$ for a *n*-qubit quantum circuit. To reduce the cost, we have proposed a new technique, namely the polynomial encoding method, inspired by functional approximation theory. We relate these polynomial bases to the multi-qubit phenomena in quantum circuits. The polynomial encoding method shows promising results with respect to gate complexity within an approximated error bound, as reported in this article. This work could be extended to non-diagonal energy operator encoding and its use in studying many body systems.

## Figures and Tables

**Figure 8:**
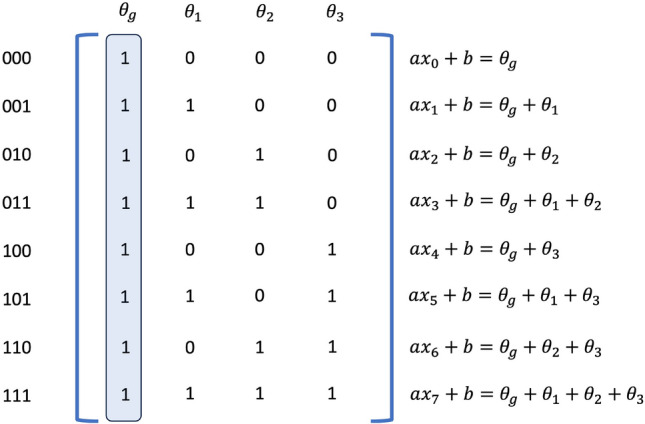
Schematic for expressing a linear function using phase gates

**Figure 9:**
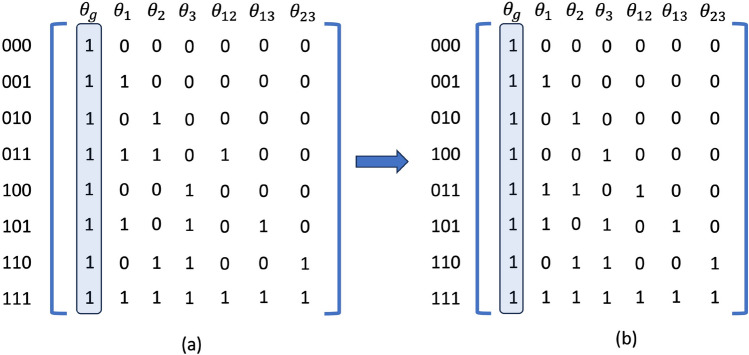
System of equations for expressing a quadratic function, original form and a lower triangular form after row exchange.

## Data Availability

The data supporting the results, discussion and conclusion in the present paper are all presented in the main manuscript and are available on request from the corresponding author.

## Appendix

### Function encoding using multi-qubit phase gates

#### Proposition 1

*To perfectly simulate a Hamiltonian (as a time evolution operator) resembling a function of degree* *r*, *using single and multi-qubit phase gates in a quantum circuit, the total number of gates required will be given by the following expression*

20 $$\begin{aligned} N_g=^nC_0 + ^nC_1 + ^nC_2 + \dots + ^nC_r. \end{aligned}$$

*In* (20), *we have an n qubit system, and the number of possible multi-qubit phase gates involving* *r* *qubits will be* $^nC_r$ (e.g., $^nC_1$—*single qubit gates*, $^nC_2$—*two-qubit gates,* $^nC_3$—*three-qubit gates, and so on*). *If we are to encode a function of degree m perfectly, the quantum system we will need must have* $n\ge m$ *qubits. If we have* $n=m$ *, the number of gates is given by*

21 $$\begin{aligned} N_g=^nC_0 + ^nC_1 + ^nC_2 + \dots + ^nC_n = 2^n. \end{aligned}$$

*In other words, we cannot encode a function of degree* $n+1$ *with* *n* *qubits*.

To prove this fact, we first look at a function of degree one, i.e., an affine (linear) function. We will need $^nC_0 + ^nC_1$ phase gates by the above proposition. Out of which $^nC_0 = 1$ phase gate is not a gate but the global phase. We also have $^nC_1$ single qubit gates to represent the function. Every time we add a phase gate, $P(\theta )$, to a qubit, all states that have that qubit in state $\mathinner {|{1}\rangle }$ get affected by an imaginary exponential of that phase parameter. For example, the phase gate with parameter $\theta _1$ acting on qubit 1 ($q_1$) will affect all the states where the first qubit (or the least significant qubit—lsqb) is in state $\mathinner {|{1}\rangle }$ - $e^{i\theta _1}\mathinner {|{001}\rangle }, e^{i\theta _1}\mathinner {|{011}\rangle }, e^{i\theta _1}\mathinner {|{101}\rangle }$ and $e^{i\theta _1}\mathinner {|{111}\rangle }$. To encode an affine function in a 3-qubit system, we will need $^3C_1 = 3$, single qubit phase gates and a global phase. The domain of the function (let us say *x*) is discretized over $2^3 = 8$ equally spaced points starting from $\mathinner {|{000}\rangle }$ to $\mathinner {|{111}\rangle }$. The function can be expressed as $y = A_1\theta$ (1 in the subscript denotes a degree one function). Figure 8 gives the schematic for expressing the function using single-qubit phase gates. The matrix $A_1$ is given within the square brackets. In the matrix, if we ignore the column corresponding to the global phase $\theta _g$, we can observe that every row of the matrix directly maps to the binary progression in the domain *x*.

Every row in the matrix $A_1$ corresponds to an equation $y_i = ax_i + b$. We can then write our function in terms of basis functions $f_{\{\theta _i\}}(x)$. The basis functions $f_{\{\theta _i\}}(x)$ takes a value 1 or a 0 depending upon whether $\theta _i$ participates in the equation or not, as given by the columns in $A_1$. For example,

$$f_{\{\theta _1\}}(x_1) = f_{\{\theta _1\}}(x_3) = f_{\{\theta _1\}}(x_5) = f_{\{\theta _1\}}(x_7) = 1,$$

whereas,

$$f_{\{\theta _1\}}(x_0) = f_{\{\theta _1\}}(x_2) = f_{\{\theta _1\}}(x_4) = f_{\{\theta _1\}}(x_6) = 0.$$

We can then write our equations in the below form

22 $$\begin{aligned} ax_i + b = \sum _{j=1}^{3} \theta _j f_{\{\theta _j\}}(x_i) + \theta _g. \end{aligned}$$

We take the example of a case where $x=\left[ 0,1,2,\ldots , 7\right]$. Since the rows of the matrix follow the binary digit progression, we will realize that the following expression

23 $$\begin{aligned} ax_i + b = 4a f_{\{\theta _3\}}(x_i) + 2a f_{\{\theta _2\}}(x_i) + a f_{\{\theta _1\}}(x_i) + b. \end{aligned}$$

Here, we see $\theta _g = b, \theta _3 = 4a, \theta _2 = 2a$ and $\theta _1 = a$. Since the rows are binary digit progressions, the columns in the matrix shown in Figure 8 are linearly independent. Further, the linear function falls within the span of the columns of the matrix. As a result, the solution will be unique. Now, let us take a look at a quadratic function. To get a quadratic equation, we can take a square of equations of the form given by (22) as

24 $$\begin{aligned} g(x_i)&= cx_i^2 + dx_i + e \nonumber \\&= \sum _{j=1, k\ge j}^{3} \theta _{jk} f_{\{\theta _j\}}(x_i) f_{\{\theta _k\}}(x_i) + \sum _{j=1}^{3}\theta _j \theta _g f_{\{\theta _j\}}(x_i) + \theta _g ^2. \end{aligned}$$

Here, we face a new set of basis functions $f_{\{\theta _j\}}(x_i) f_{\{\theta _k\}}$. We will call these basis functions as $f_{\{\theta _{jk\}}}(x_i)$. These basis functions take the value 1 only when both $f_{\{\theta _j\}}(x_i)$ and $f_{\{\theta _k\}}(x_i)$ take the value 1. This can be associated with a two-qubit phase gate. Two qubit phase gates act on all those states where both the qubits are in state $\mathinner {|{1}\rangle }$. A new set of parameters given by $\theta _{jk}$ will also be needed. These will be proportional to the two-qubit phase gate parameters. We will have cases where $j=k$ in equation (24). This will translate to terms like $f_{\{\theta _j\}}^2(x_i)$. Since the basis functions only take values 0 and 1, we will have $f_{\{\theta _j\}}^2(x_i) = f_{\{\theta _j\}}(x_i)$. The parameters for this basis function can be rewritten as $\theta _j$. These will correspond to the single qubit phase gates. (24) can be re-written as follows

25 $$\begin{aligned} \begin{aligned} g(x_i)&= \sum _{j=1}^3 (\theta _{j} + \theta _g) f_{\{\theta _j\}}(x_i) + \sum _{j=1, k>j}^{3} \theta _{jk} f_{\{\theta _j\}}(x_i) f_{\{\theta _k\}}(x_i) + \theta _g ^2 \\&= \sum _{j=1}^3 \theta _{j}' f_{\{\theta _j\}}(x_i) + \sum _{j=1, k>j}^{3} \theta _{jk} f_{\{\theta _j\}}(x_i) f_{\{\theta _k\}}(x_i) + \theta _g'. \end{aligned} \end{aligned}$$

We have a new set of single qubit phase parameters given by $\theta _j'$ and a new global phase given by $\theta _g'$. The number of two-qubit gate parameters needed is $^3C_2$. The total number of needed parameters is given by $^3C_0 + ^3C_1 + ^3C_2$. The term $^3C_0 = 1$ corresponds to the global phase. $^3C_1$ denote the number of single qubit phase gates, and $^3C_2$, the number of two qubit phase gates.

The system of equations corresponding to the quadratic function can be given by the matrix shown in Fig. 9a. We will call this matrix $A_2$. The matrix in Fig. 8 (matrix $A_1$) is a subset of the matrix $A_2$, as the first four columns are the same in both of them, $A_1 \subset A_2$. The three extra columns in $A_2$ bring the extra information required to encode a quadratic function. The matrix $A_2$ can be converted to a lower triangular form by a suitable exchange of rows, as shown in Fig. 9b. This proves that the columns of matrix $A_2$ are linearly independent. The solution to the quadratic function is thus unique as the function lies within the span of the columns of $A_2$ as given in Eq. (25).

#### *k*th degree function

We encode the function for a $k^{th}$ degree function in an *n* ($n\ge k$) qubit system as

26 $$\begin{aligned} g(x_i) = \sum _{j_1 = 1, j_2 \ge j_1, j_3 \ge j_2, \ldots , j_k \ge j_{k-1}} ^n \theta _{j_1j_2\ldots j_k} f_{\{\theta _{j_1}\}}(x_i) f_{\{\theta _{j_2}\}}(x_i)\ldots f_{\{\theta _{j_k}\}}(x_i) + \theta _g. \end{aligned}$$

Here, $f_{\{\theta _{j_r}\}}(x) \in \{0,1\}$, for any $r \in \left[ 1,k\right]$, we have $f_{\{\theta _{j_r}\}}^m(x) = f_{\{\theta _{j_r}\}}(x)$ for all $m \in \mathbb {Z}$. As such, all terms where $j_1 = j_2 = \ldots = j_k = p ~(p\in \left[ 1, n\right] )$ will correspond to single qubit phase gates. Such terms will be $^nC_1 = n$, with a parameter $\theta _p$. Then, we will have terms where $j_1 = j_2 = \ldots = j_{k-1} = p, j_k > p$. These will correspond to two-qubit phase gates with parameters $\theta _{pj_k}$. There will be $^nC_2$ such terms. Going further, we will next have terms where $j_1 = j_2 = \ldots = j_{k-2} = p, j_{k-1}> p, j_k > j_{k-1}$. These will correspond to three-qubit phase gates with parameters $\theta _{pj_{k-1}j_k}$. There will be $^nC_3$ such terms. We can go on with this progression till we reach terms like $j_1 = p, j_2> j_1, j_3>j_2, \ldots j_k > j_{k-1}$. These will correspond to *k* qubit phase gates with parameters $\theta _{pj_2 \ldots j_{k-1}j_k}$. There are $^nC_k$ such terms. It points to the fact that to encode a $k^{th}$ degree function, we will need *k* qubit phase gates. The total number of parameters needed is $^nC_0 + ^nC_1 + \ldots ^nC_k$.

#### Encoding limitations

While we can encode a *k*th degree function in a *n* ( with $n\ge k$) qubit system, we cannot do so in a system where $n<k$. We encode a *k*th degree function in a *k* qubit system. The total number of parameters needed would be $^kC_0 + ^kC_1 + \ldots ^kC_k = 2^k$. If we call the matrix representing the system of equations as $A_k$, we have $A_k \in \mathbb {R}^{2^k \times 2^k}$. The number of columns equals the number of rows as $2^k$ discretized domain points are in a *k* qubit system. As shown before, in a three-qubit system, the columns are all independent, and it is a full-rank invertible matrix. The solution for the *k*th degree function is thus unique. If we are to encode a function of degree $k+1$, we will need to add extra parameters and, thus, extra columns to this matrix. Since the rank of this matrix cannot exceed $2^k$, any columns we add to $A_k$ will not add any extra information as these columns will be linearly dependent on the existing columns. Moreover, if we consider a $(k+1)$ qubit system for encoding a function of degree $(k+1)$, the matrix will be $A_{k+1} \in \mathbb {R}^{2^{(k+1)} \times 2^{(k+1)}}$. The solution that this matrix will offer to encode a function of degree $(k+1)$ will also be unique with $2^{(k+1)}$ independent columns. Now, by proper ordering of the columns of $A_{k+1}$, we can show that the first $2^k \times 2^k$ principal minor of $A_{k+1}$ is $A_k$, i.e., $A_k \subset A_{k+1}$. Since $A_{k+1}$ is already providing a unique solution to a $(k+1)$th degree function, we cannot get a unique solution from $A_k$.

### Table of simulation parameters in $ibm\_auckland$ hardware

We have performed experiments on an IBM quantum machine, namely $ibm\_auckland$. A snapshot of the simulation parameters, including decoherence time parameters ($T_1$, and $T_2$), read-out error, CNOT gate error (while its connections are also shown), and different gate timing is given in Table 7.

**Table 7:** A snapshot of simulation parameters obtained in $ibm\_auckland$ quantum machine.

Qubit	$T_1$ ($\upmu$s)	$T_2$ ($\upmu$s)	Frequency (GHz)	Readout error	CNOT connection	CNOT error	Gate time (ns)
0	56.46468	141.7933	4.9329813	0.0091	0_1	0.005677	469.33333
1	191.69290	196.7209	5.0738154	0.0099	1_4	0.005926	497.77777
2	132.8908	132.8376	5.0057532	0.0077	2_3	0.010415	355.55555
3	184.30230	104.5629	4.8972571	0.0075	3_5	0.005733	320
4	232.67380	214.8679	4.9201675	0.0071	4_7	0.007218	483.55555
5	194.22661	49.1762	4.9928249	0.0089	5_8	0.005188	362.66666
6	142.48602	154.5315	5.0067022	0.0108	6_7	0.008608	490.66666
7	172.24152	172.5634	4.8284356	0.0069	7_10	0.022260	433.77777
8	115.51685	76.1907	5.2035975	0.0109	8_11	0.005506	369.7777
$\vdots$	$\vdots$	$\vdots$	$\vdots$	$\vdots$	$\vdots$	$\vdots$	$\vdots$

## Code availibility

The code developed in this study is available upon reasonable request from the corresponding author.
